# Characterization of Polysaccharide Extracts of Four Edible Mushrooms and Determination of *InVitro* Antioxidant, Enzyme Inhibition and Anticancer Activities

**DOI:** 10.1021/acsomega.4c00322

**Published:** 2024-06-06

**Authors:** Ebru Deveci, Gülsen Tel-Çayan, Fatih Çayan, Bahar Yılmaz Altınok, Sinan Aktaş

**Affiliations:** † Chemistry and Chemical Processing Technology Department, Technical Sciences Vocational School, Konya Technical University, Konya 42100, Turkey; ‡ Department of Chemistry and Chemical Processing Technologies, Muğla Vocational School, Muğla Sıtkı Koçman University, Muğla 48000, Turkey; § Department of Bioengineering, Faculty of Engineering, Karamanoğlu Mehmetbey University, Karaman 70000, Turkey; ∥ Department of Biology, Faculty of Science, Selçuk University, Konya 42100, Turkey

## Abstract

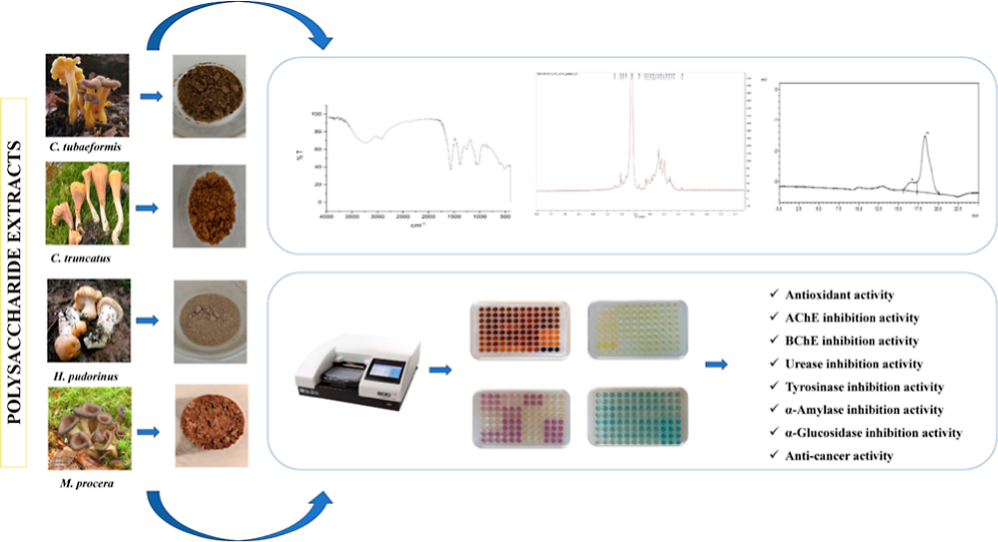

Mushroom polysaccharides are important bioactive compounds derived from mushrooms with various beneficial properties. In this study, the chemical characterization and bioactivities of polysaccharide extracts from four different edible mushrooms, *Clavariadelphus truncatus* Donk, *Craterellus tubaeformis* (Fr.) Quél., *Hygrophorus pudorinus* (Fr.) Fr., and *Macrolepiota procera* (Scop.) Singer were studied. Glucose (13.24–56.02%), galactose (14.18–64.05%), mannose (2.18–18.13%), fucose (1.21–5.78%), and arabinose (0.04–5.43%) were identified in all polysaccharide extracts by GC-MS (gas chromatography–mass spectrometry). FT-IR (Fourier transform infrared spectroscopy) confirmed the presence of characteristic carbohydrate patterns. ^1^H NMR suggested that all polysaccharide extracts had α- and β-d-mannopyranose, d-glucopyranose, d-galactopyranose, α-l-arabinofuranose, and α-l-fucopyranose residues. Approximate molecular weights of polysaccharide extracts were determined by HPLC (high-performance liquid chromatography). The best antioxidant activity was found in *M. procera* polysaccharide extract in DPPH^•^ (1,1-diphenyl-2-picrylhydrazyl) scavenging (39.03% at 800 μg/mL), CUPRAC (cupric reducing antioxidant capacity) (A_0.50_: 387.50 μg/mL), and PRAP (phosphomolybdenum reducing antioxidant power) (A_0.50_: 384.08 μg/mL) assays. *C. truncatus* polysaccharide extract showed the highest antioxidant activity in ABTS^•+^ scavenging (IC_50_: 734.09 μg/mL), β-carotene-linoleic acid (IC_50_: 472.16 μg/mL), and iron chelating (IC_50_: 180.35 μg/mL) assays. Significant anticancer activity was found in *C. truncatus* polysaccharide extract on HT-29 (IC_50_: 46.49 μg/mL) and HepG2 (IC_50_: 48.50 μg/mL) cell lines and *H. pudorinus* polysaccharide extract on the HeLa cell line (IC_50_: 51.64 μg/mL). Also, *H. pudorinus* polysaccharide extract possessed prominent AChE (acetylcholinesterase) inhibition activity (49.14% at 200 μg/mL).

## 1 Introduction

Mushrooms have a valuable place in the food, health, pharmaceutical, and cosmetic industries due to their unique taste and extraordinary nutritional and medicinal values. Today, it is known that there are 110.000 mushroom species in the world, and only 10% of these mushrooms have been officially described.^1^ Edible mushrooms are functional foods and are used as dietary supplements because they contain bioactive compounds such as proteoglycans, polysaccharides, glycoproteins, phenolics, terpenoids, vitamins, alkaloids, sterols, lactones, and nucleotide analogs.^2^ Polysaccharides are essential components of edible mushrooms and have attracted much attention due to their bioactivities such as antioxidant, immunomodulator, anticancer, hypoglycemic, and hypolipidemic. Especially in the last few decades, mushroom polysaccharides have displayed extraordinary potential in various disciplines, including immunology, molecular biology, pharmaceutical chemistry, and biotechnology.^3,4^

The increasing cancer burden worldwide highlights the need for improving current therapeutic strategies. Cancer, which has 36 different types, generally affects men as lung (1.44 million new cases and 1.18 million deaths), prostate (1.42 million new cases and 374,000 deaths), colon (1.07 million new cases with 512,000 deaths), stomach (717,000 new cases and 501,000 deaths), and liver (636,000 new cases with 578,000 deaths) cancers, and women as breast (2.25 million new cases and 682,000 deaths), colon (931,000 new cases with 418,000 deaths), lung (773,000 new cases with 603,000 deaths), cervical (598,000 new cases with 339,000 deaths), and stomach (368,000 new cases with 260,000 deaths) cancers in 2020.^5^ Cancer therapy has been an ever-evolving field of research for several decades, and there are many modern as well as traditional techniques exerted against cancer. Various techniques for cancer treatment can be listed as chemotherapy, radiation therapy, or surgery. However, they all have disadvantages.^6^ The use of conventional chemicals has side effects and toxicities. Especially due to the limitations of traditional chemotherapeutic approaches, the need for new approaches for the control of cancer is increasing. For this purpose, there is an increasing need for new tactics for the prevention or treatment of cancer to control the mortality rate.^7^

Disruption of the balance between the formation of reactive oxygen/nitrogen species (ROS/RNS) and the cellular antioxidant defense system results in oxidative stress. Increased oxidative stress causes notable damage to biological systems, involving molecular damage (such as nucleic acids, lipids, and proteins) that can seriously affect health. Oxidative stress-based damage to biomolecules or stimulation of several secondary reactive species results in cell death (apoptosis or necrosis).^8^ ROS were reported to cause high metastasis, radioresistance, and carcinogenesis in cancer.^9^ ROS have negative effects on diabetes by improving insulin resistance through negative regulation of insulin signaling.^10^ Excessive ROS production has implications for Alzheimer’s disease through the accumulation of β-amyloid plaques in the brain by triggering pro-inflammatory signaling, apoptosis, and necrosis.^11^ Oxidative stress has been estimated to be related to more than 100 diseases, such as hypertension, cardiovascular disease, cancer, diabetes, and neurodegenerative diseases. Antioxidants prevent or abolish oxidative stress-related diseases by counteracting the aggravating effects of ROS/RNS.^12^ Antioxidants scavenge free radicals and play a critical role in maintaining optimal cellular functions. Laboratory, animal, and human observation studies confirmed that both dietary supplements and endogenous antioxidants prevent tumor development and progression by neutralizing ROS. It has been proven that a high intake of antioxidant-rich foods is inversely associated with cancer risk.^13,14^ Many natural and synthetic antioxidants have been introduced, but their toxic effects have been proven.^15^ For these reasons, the tendency to obtain compounds with antioxidant potential from natural products is constantly increasing.

Enzymes, which have a leading role in numerous catalytic reactions, also lead to negative effects on food spoilage or human health with their biocatalytic capacities.^16^ Enzyme inhibitors dominate the activities of respective enzymes via interacting with the active site. In this context, enzyme inhibitors are an important part of the clinical drug class. Enzyme inhibition is an essential approach and accounts for nearly half of all marketed small-molecule drugs.^17^ For example, cholinesterase inhibitors are valued as therapeutics in the management of Alzheimer’s disease, tyrosinase inhibitors in melanoma, and α-glucosidase and α-amylase inhibitors in diabetes.^18^

*Clavariadelphus truncatus* Donk (club coral mushroom) is an edible mushroom with high nutritional value. It has been reported that clavaric acid (isolated from *C. truncatus*) can be used in the treatment of some cancers by interacting with farnesyltransferase, which is effective in tumor formation.^19^ The antioxidant, enzyme inhibition, antimicrobial, and anticancer properties of many varieties of extracts obtained from this mushroom have also been examined.^20,21^*Craterellus tubaeformis* (Fr.) Quél. (funnel mushroom) is a popular edible mushroom. Three polysaccharides from *C. tubaeformis* consisting of →2,6)-α-man-(1→ and →6)-α-Gal-(1→ chains, →6)-β-Glc-(1→, with branches of single β-Glc residues or short →3)-β-Glc-(1→ chains were previously reported.^22^ The hexane, methanol, and water extracts of *C. tubaeformis* were investigated for antioxidant, anticancer, and anti-enzyme activities with chemical characterization by high-performance liquid chromatography (HPLC).^20^*Hygrophorus pudorinus* (Fr.) Fr. (waxcap mushroom) is an edible mushroom. To our knowledge, the only study on the bioactivities and chemical composition of *H. pudorinus* extracts reported that this mushroom was rich in fumaric acid and had antioxidant, anti-AChE, and antidiabetic activities.^20^*Macrolepiota procera* (Scop.) Singer (parasol mushroom) is a popular edible mushroom. Neutral branched (acetylated) β-d-glucomannan and α-1,4-d-glucan polysaccharides with anti-immunomodulatory and antibacterial activities were purified from *M. procera*.^23^ Also, phenolic and triterpene compounds and antioxidant, antiproliferative, and anticholinesterase activities of different extracts of this mushroom were investigated in earlier studies.^24,25^

In recent years, the remarkable bioactive properties of mushrooms have been attributed to their polysaccharide contents, and interest in the study of polysaccharides has increased. When no studies have been reported on *H. pudorinus* and *C. truncatus* polysaccharides, there are limited number of studies on *C. tubaeformis* and *M. procera* polysaccharides. Therefore, this study aimed to address the chemical characterization and bioactivities of polysaccharide extracts from four different edible mushrooms, namely, *C. truncatus* Donk, *H. pudorinus* (Fr.) Fr., *C. tubaeformis* (Fr.) Quél., and *M. procera* (Scop.) Singer. Chemical characterization of the polysaccharide extracts was carried out by using gas chromatography–mass spectrometry (GC–MS), Fourier transform infrared spectroscopy (FT-IR), ^1^H NMR, and HPLC analyses. Total carbohydrate and total protein contents of the polysaccharide extracts were measured. Additionally, the antioxidant, enzyme inhibition, and anticancer activities of the polysaccharide extracts were investigated.

## 2 Results and Discussion

### 2.1 Total Carbohydrate and Total Protein Contents

Total carbohydrate and total protein contents of the polysaccharide extracts were tested according to the phenol-sulfuric acid and Bradford methods, respectively, and the results are depicted in Table 1. Total carbohydrate contents of *C. tubaeformis*, *C. truncatus*, *H. pudorinus*, and *M. procera* polysaccharide extracts were calculated as, respectively: 77.96 ± 1.10, 64.93 ± 0.98, 73.22 ± 2.14, and 69.37 ± 1.50%. Total protein contents of *C. tubaeformis*, *C. truncatus*, *H. pudorinus*, and *M. procera* polysaccharide extracts were calculated as respectively: 0.78 ± 0.25, 2.85 ± 0.87, 0.41 ± 0.05, and 3.78 ± 0.96%. In accordance with the obtained results, total carbohydrate contents of Crat HW1, Crat 2%1, and Crat 25%1 polysaccharide extracts of *C. tubaeformis* (from Finland) were reported as 73.4 ± 2.0, 87.9 ± 5.5, and >95%, respectively, while total protein contents were noted as 31.6 ± 1.6, <2, and <2%, respectively.^22^ Georgiev et al. determined similar amounts of total carbohydrate (74.1 ± 0.7%) and higher amounts of total protein (12.7 ± 0.2%) contents in *M. procera* (from Bulgaria) polysaccharide extract than our results.^23^ As different from our findings, the total carbohydrate content of the *Craterellus cornucopioides* (a different member of *Craterellus*) polysaccharide fraction was reported as 99.15% with no total protein.^26^

**Table 1:** Total Carbohydrate and Total Protein Contents of Polysaccharide Extracts^a^

polysaccharide extracts	total carbohydrate content (%)	total protein content (%)
*C. tubaeformis*	77.96 ± 1.10	0.78 ± 0.25
*C. truncatus*	64.93 ± 0.98	2.85 ± 0.87
*H. pudorinus*	73.22 ± 2.14	0.41 ± 0.05
*M. procera*	69.37 ± 1.50	3.78 ± 0.96

a Values represent the means ± SE of three parallel sample measurements (*p* < 0.05).

### 2.2 Monosaccharide Composition

The monosaccharide compositions of the polysaccharide extracts were identified by GC-MS, and the results are given in Table 2. Glucose, galactose, mannose, fucose, and arabinose were identified in all four polysaccharide extracts. *C. tubaeformis* polysaccharide extract was mainly composed of glucose (56.02%), mannose (18.13%), and galactose (14.18%). The prominent monosaccharides were identified as glucose (42.17%), galactose (28.12%), and mannose (14.19%) in *C. truncatus* polysaccharide extract. *M. procera* polysaccharide extract had mainly galactose (64.05%) and glucose (17.13%). Galactose (60.81%), mannose (17.63%), and glucose (13.24%) were found as the main monosaccharides in *H. pudorinus* polysaccharide extract. The polysaccharides with high amounts of glucose, galactose, and mannose from *Craterellus* and *Macrolepiota* mushroom species have been isolated in prior studies. Consistent with our results, it was determined that *M. procera* (from Bulgaria) polysaccharide extract contained high amounts of glucose (62.3%), galactose (19.7%), mannose (6.9%), and minor amounts of fucose (3.4%).^23^ The main monosaccharides of Crat HW1, Crat 2%1, and Crat 25%1 polysaccharide extracts from *C. tubaeformis* (from Finland) were reported as glucose (11.9 ± 0.5–68.5 ± 0.5%), galactose (4.1 ± 0.2–17.8 ± 0.6%), mannose (17.8 ± 0.1–32.6 ± 0.6%), and xylose (5.7 ± 0.0–22.0 ± 0.3%).^22^ The monosaccharide composition of *C. cornucopioides* polysaccharide fraction was mannose: xylose: glucose: fructose: arabinose with a molar ratio of 0.7:0.18:0.05:0.05:1.^26^ In a different study on *C. cornucopioides* polysaccharide extract, the monosaccharide composition was detected as xylose: glucose: galactose with a 2:5:4 molar ratio.^27^

**Table 2:** Monosaccharide Composition of the Polysaccharide Extracts^a^

monosaccharides	retention time (min)	*C. tubaeformis* (%)	*C. truncatus* (%)	*H. pudorinus* (%)	*M. procera* (%)
arabinose	14.05	0.48	0.04	0.23	5.43
rhamnose	14.50	0.34	0.15	Nd	Nd
fucose	14.75	1.21	5.78	1.33	3.22
xylose	15.19	6.28	3.26	Nd	Nd
mannose	16.13	18.13	14.49	17.63	2.18
galactose	17.08	14.18	28.12	60.81	64.05
glucose	17.52	56.02	42.17	13.24	17.13

a Nd: not detected.

### 2.3 FT-IR Analysis

**Figure 1:**
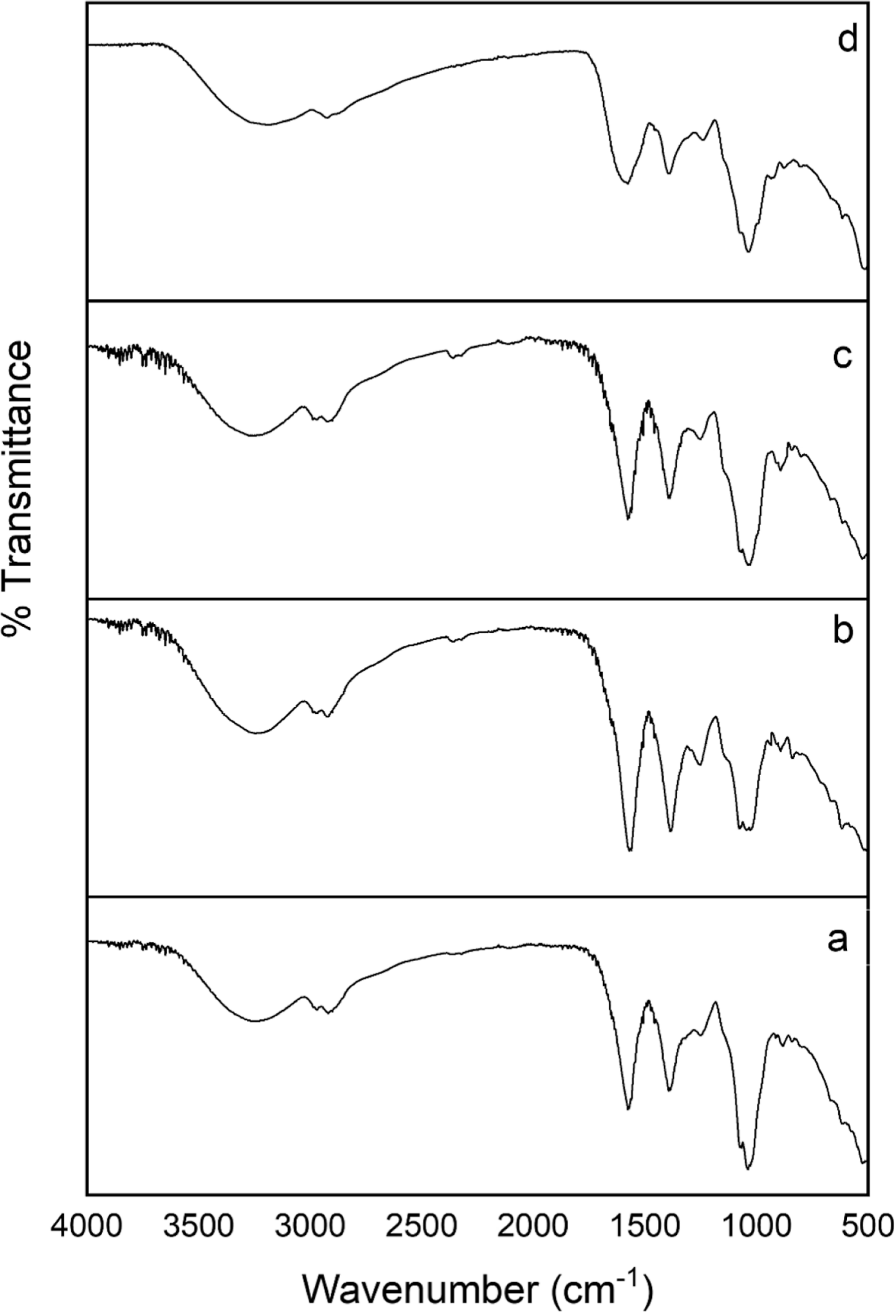
FT-IR spectra of the polysaccharide extracts (a) *C. tubaeformis* (b) *C. truncatus* (c) *H. pudorinus* (d) *M. procera*.

The typical carbohydrate patterns were observed in FT-IR spectra of four polysaccharide extracts as seen in Figure 1. The assignments of FT-IR absorption bands are given in Table 3. The intense absorption bands around 3200 cm^–1^ were attributed to O–H stretching. The weak absorption bands around 2922 cm^–1^ belonged to C–H stretching in the pyranose ring. The strong overlapping bands around 1100–1030 cm^–1^ represented the typical polysaccharide backbone pattern corresponding to C–O–C (glycosidic) and C–O bonds stretching. The bands around 1575 cm^–1^ were caused by C=O stretching of the protein amides. The bands around 1390 cm^–1^ were attributed to −CH (O–CH_2_) stretching. The weak bands around 1250 cm^–1^ proved the presence of C–O stretching. The characteristic bands >900 cm^–1^ suggested the existence of α-glycosidic linkage of the sugar units. The characteristic bands around 880 cm^–1^ showed the presence of β-glycosidic linkage of the sugar units.^22,23,28,29^

**Table 3:** Assignments of FT-IR Absorption Bands

	wavenumber (cm^–1^)			
assignments	*C. tubaeformis*	*C. truncatus*	*H. pudorinus*	*M. procera*
O–H stretching	3244	3244	3244	3196
C–H stretching in pyranose ring	2921	2922	2922	2924
C–O and C–O–C (glycosidic) stretching	1066–1037	1075–1026	1060–1036	1100–1035
C=O stretching	1575	1568	1575	1575
–CH (O–CH_2_) stretching	1393	1385	1394	1393
C–O stretching	1250	1251	1250	1239
C–H stretching in α-glycosidic linkage	912	910	908	934
C–H stretching in β-glycosidic linkage	880	890	888	876

### 2.4 ^1^H NMR Analysis

**Figure 2:**
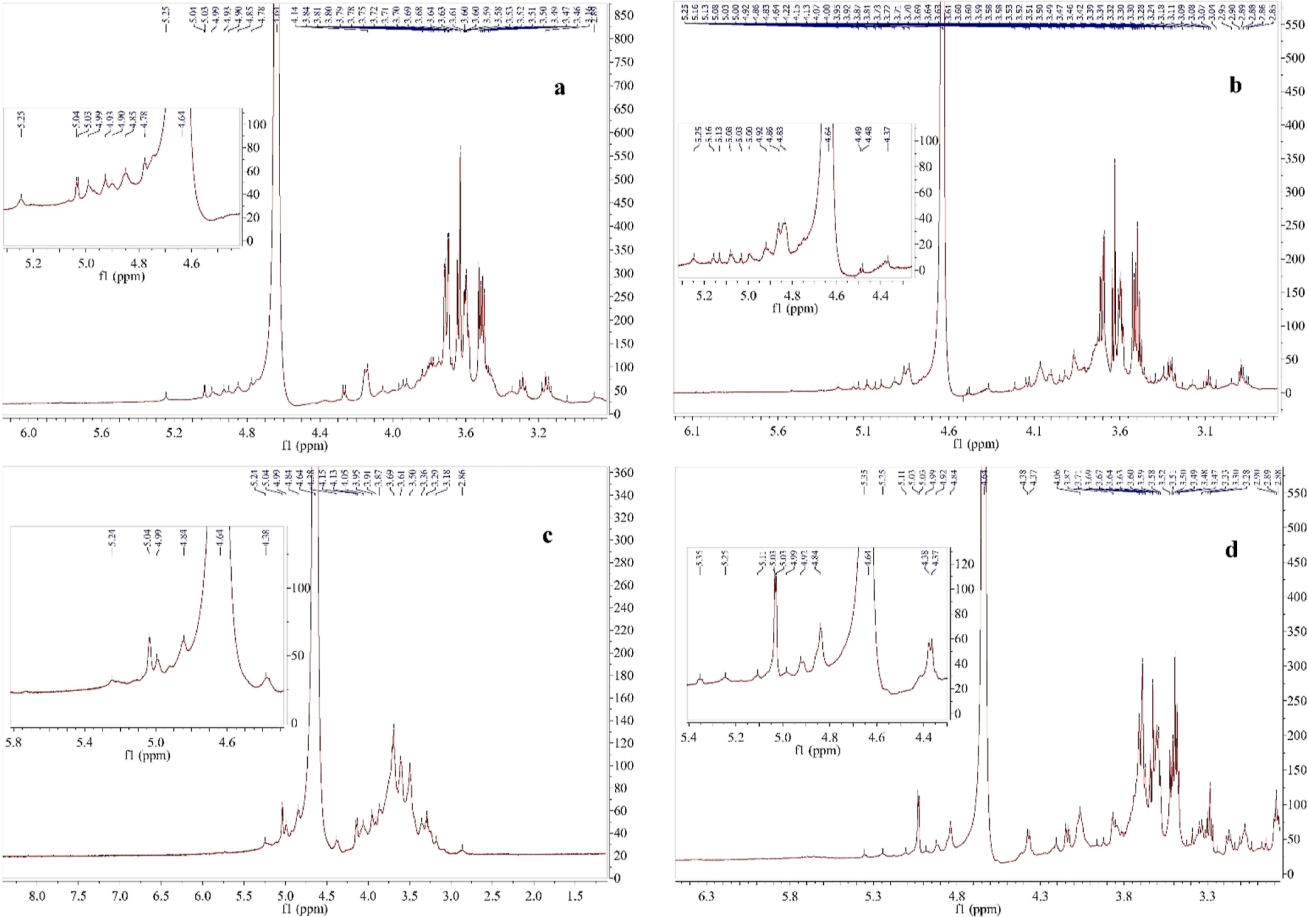
Full ^1^H NMR spectra of the polysaccharide extracts (a) ^1^H NMR spectrum of *C. tubaeformis* polysaccharide extract. (b) ^1^H NMR spectrum of *C. truncatus* polysaccharide extract. (c) ^1^H NMR spectrum of *H. pudorinus* polysaccharide extract. (d) ^1^H NMR spectrum of *M. procera* polysaccharide extract. Chemical shifts expressed in ppm.

^1^H NMR was used to determine characteristic proton interactions in the polysaccharide extracts, and the ^1^H NMR spectra of the polysaccharide extracts are shown in Figure 2. ^1^H NMR spectra showed that four polysaccharide extracts had typical carbohydrate patterns at δ 3.0 to δ 5.4 ppm.^30^^1^H NMR suggested that all polysaccharide extracts had α- and β-d-mannopyranose, d-glucopyranose, d-galactopyranose, α-l-arabinofuranose, and α-l-fucopyranose residues.

Eight anomeric proton signals were identified in the ^1^H NMR spectrum of *C. tubaeformis* polysaccharide extract. The signal at δ 5.25 ppm could be referred to as α-d-glucopyranose or α-l-fucopyranose; the signals at δ 5.04, δ 5.03, and δ 4.99 ppm to α-d-galactopyranose or α-d-mannopyranose or α-l-arabinofuranose or α-l-rhamnopyranose; the signals at δ 4.93, δ 4.90, and δ 4.85 ppm to β-d-mannopyranose or α-d-xylopyranose; the signal at δ 4.78 ppm to β-d-glucopyranose. The overlapped signals between δ 3.04–4.14 ppm indicated H2–H6 protons in each sugar unit.^29−37^

Twelve anomeric proton signals were identified in the ^1^H NMR spectrum of *C. truncatus* polysaccharide extract. The signal at δ 5.25 ppm could be assigned to α-d-glucopyranose or α-l-fucopyranose; the signals at δ 5.16, δ 5.13, δ 5.08, δ 5.03, and δ 5.00 ppm to α-d-galactopyranose or α-d-mannopyranose or α-l-arabinofuranose or α-l-rhamnopyranose; the signals at δ 4.92, δ 4.86, and δ 4.83 ppm to β-d-mannopyranose or α-d-xylopyranose; the signals at δ 4.48 and δ 4.49 ppm to β-d-glucopyranose or β-d-xylopyranose; the signal at δ 4.37 ppm to terminal β-d-galactopyranose. The overlapped signals between δ 3.01–4.22 ppm suggested the presence of H2–H6 protons in each sugar unit.^29−37^

Five anomeric proton signals were identified in the ^1^H NMR spectrum of *H. pudorinus* polysaccharide extract. The signal at δ 5.24 ppm could be attributed to α-d-glucopyranose or α-l-fucopyranose; the signals at δ 5.04 and δ 4.99 ppm to α-d-galactopyranose or α-d-mannopyranose or α-l-arabinofuranose; the signal at δ 4.84 ppm to β-d-mannopyranose; the signal at δ 4.38 ppm to terminal β-d-galactopyranose or substituted β-d-galactopyranose. The overlapped signals between δ 3.18–4.15 ppm concern H2–H6 protons in each sugar unit.^29−37^

Nine anomeric proton signals were identified in the ^1^H NMR spectrum of *M. procera* polysaccharide extract. The signal at δ 5.35 ppm may be the characteristic proton signal of →4)-α-d-glucopyranosyl-(1→. The signal at δ 5.25 ppm could be assigned to α-d-glucopyranose or α-l-fucopyranose; the signals at δ 5.11, δ 5.03, and δ 4.99 ppm to α-d-galactopyranose or α-d-mannopyranose or α-l-arabinofuranose; the signals at δ 4.92 and δ 4.84 ppm to β-d-mannopyranose; the signals at δ 4.38 and δ 4.37 ppm to terminal β-d-galactopyranose or substituted β-d-galactopyranose. The overlapped signals between δ 3.17–4.21 ppm concern H2–H6 protons in each sugar unit.^29−37^

### 2.5 Approximate Molecular Weight

**Figure 3:**
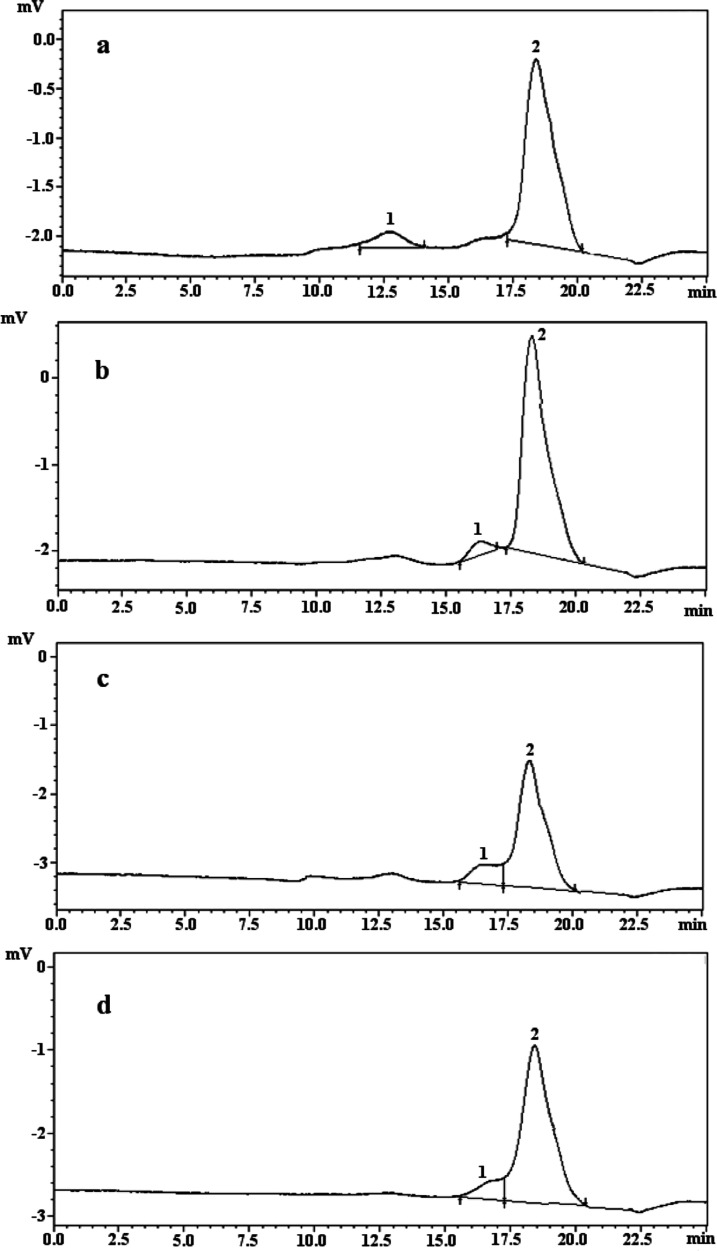
HPLC chromatograms of the polysaccharide extracts (a) (1) 7.83 × 10^5^ and (2) 2.86 × 10^3^ Da in *C. tubaeformis* polysaccharide extract. (b) (1) 1.4 × 10^4^ and (2) 2.27 × 10^3^ Da in *C. truncatus* polysaccharide extract. (c) (1) 1.46 × 10^4^ and (2) 3.09 × 10^3^ Da in *H. pudorinus* polysaccharide extract. (d) (1) 1.21 × 10^4^ and (2) 3.21 × 10^3^ Da in *M. procera* polysaccharide extract.

The approximate molecular weights (MWs) of the polysaccharide extracts were determined by HPLC. The HPLC chromatograms of the polysaccharide extracts are shown in Figure 3. Two different polysaccharides with approximate MWs of 2.86 × 10^3^ and 7.83 × 10^5^ Da were identified in *C. tubaeformis* polysaccharide extract; 2.27 × 10^3^ and 1.4 × 10^4^ Da in *C. truncatus* polysaccharide extract; 3.09 × 10^3^ and 1.46 × 10^4^ Da in *H. pudorinus* polysaccharide extract; 3.21 × 10^3^ and 1.21 × 10^4^ Da in *M. procera* polysaccharide extract. In a previous study, three different polysaccharide extracts were obtained from *C. tubaeformis* (from Finland) by using deionized water refluxing (Crat HW1), 2% KOH solution (Crat 2%1), and 25% KOH refluxing (Crat 25%1) methods. The approximate MWs of these polysaccharide extracts were noted as 3.96 × 10^5^, 5.08 × 10^5^, and 5.42 × 10^5^ Da, respectively.^22^ The approximate MWs of polysaccharide extracts obtained from *C. cornucopioides* were reported in detail. Two different polysaccharides were extracted from *C. cornucopioides* with the ethanol precipitation from the hot water extract method, and the approximate MWs were revealed as 1.38 × 10^5^ and 2.73 × 10^5^ Da.^30^ The average MW of the polysaccharide extract of *C. cornucopioides* according to the ethanol precipitation from the hot water extract method was found to be 8.28 × 10^4^ Da.^37^ The approximate MW of *C. cornucopioides* polysaccharide fraction (obtained by the ethanol precipitation from the hot water extract method) was reported as 9.2 × 10^5^ Da.^26^*M. procera* (from Bulgaria) polysaccharide extract was purified with a double extraction method from alcohol-insoluble parts of the mushroom, and the approximate MW was reported as 66.3 × 10^4^ g/mol.^23^ The approximate MW of the polysaccharide extract from *M. procera* (from China) obtained by the ethanol precipitation from hot water extract method was expressed as 7.71 × 10^5^ Da.^38^ Qu et al. calculated the approximate MW of *Hygrophorus lucorum* polysaccharide extract (obtained by the ethanol precipitation from the hot water extract method) as 20.4 kDa.^39^ The main reason for this discrepancy in the approximate MWs of polysaccharide extracts between the results in the literature and our research may be due to the differences in mushroom species and extraction methods or applications.^30^

### 2.6 Antioxidant Activity

Antioxidants consist of different classes according to their features, sources, polarities, and mechanisms. Since antioxidants have diverse mechanisms of action, it is recommended to apply multiple methods to determine antioxidant activity more comprehensively. Therefore, the antioxidant activities of polysaccharide extracts were investigated by β-carotene-linoleic acid, ABTS^•+^ scavenging, phosphomolybdenum reducing antioxidant power (PRAP), DPPH^•^ scavenging, cupric reducing antioxidant capacity (CUPRAC), and iron chelating assays, and the results are presented in Table 4. *M. procera* polysaccharide extract displayed the highest antioxidant activity in DPPH^•^ scavenging (39.03 ± 0.49% inhibition at 800 μg/mL), CUPRAC (A_0.50_: 387.50 ± 0.25 μg/mL), and PRAP (A_0.50_: 384.08 ± 0.02 μg/mL) assays. *C. truncatus* polysaccharide extract was found to be the most antioxidant active in ABTS^•+^ scavenging (IC_50_: 734.09 ± 0.43 μg/mL), β-carotene-linoleic acid (IC_50_: 472.16 ± 0.81 μg/mL), and iron chelating (IC_50_: 180.35 ± 0.59 μg/mL) assays. On the other hand, IC_50_ values of the standards butylated hydroxyanisole (BHA) and α-tocopherol were 1.34 ± 0.04 and 2.10 ± 0.08 μg/mL in β-carotene-linoleic acid assay; 11.76 ± 0.09 and 38.46 ± 0.54 μg/mL in the ABTS^•+^ scavenging assay, respectively. The A_0.50_ values of the standards BHA and α-tocopherol were 24.42 ± 0.69 and 89.47 ± 0.87 μg/mL in the CUPRAC assay, while the A_0.50_ value of the standard ascorbic acid was 13.66 ± 0.01 μg/mL in the PRAP assay. The standards BHA and α-tocopherol inhibited 97.79 ± 0.08 and 96.12 ± 0.42% of DPPH^•^ at 800 μg/mL, respectively. The IC_50_ value of the standard ethylenediaminetetraacetic acid (EDTA) was 3.47 ± 0.14 μg/mL in the iron chelating assay. The antioxidant activities of the studied polysaccharide extracts were found to be lower compared to the standards. The biological properties of polysaccharides vary depending on their conformation, structural composition, and the type of glycosidic linkage.^40^ In addition, studies have revealed that extraction and processing methods significantly affect the structure and conformation of the polysaccharides, thus changing their chemical and biological properties. It has been shown that there is a correlation between the MWs of polysaccharides and their antioxidant activities.^41,42^ GLPL1 and GLPL2 encoded polysaccharide extracts were isolated from *Ganoderma lucidum* in the study of Liu et al., and GLPL1 polysaccharide extract (78.3% inhibition at 0.63 mg/mL for hydroxyl radical scavenging activity, 50% inhibition at 8 mg/mL for H_2_O_2_ scavenging activity, 5.2–58% inhibition at 1.5–10 mg/mL for iron chelating activity) was reported to have higher antioxidant activity than GLPL2 (53.6% inhibition at 0.63 mg/mL for hydroxyl radical scavenging activity, 30% inhibition at 8 mg/mL for H_2_O_2_ scavenging activity, 4.2–21% inhibition at 1.5–10 mg/mL for iron chelating activity) associated with lower molecular weight.^43^ Again, in this study, it was explained by the fact that higher antioxidant activity can provide freer and thus more active hydroxyl groups at lower molecular weights compared to higher molecular weights on the same weight basis.^43^ It was emphasized that the antioxidant activities of two different polysaccharide extracts obtained from *Ganoderma leucocontextum* with codes of GLP-1 (IC_50_: 0.56, 1.32, and 0.76 mg/mL for ABTS^•+^, hydroxyl radical, and superoxide anion radical scavenging assays, respectively) and GLP-2 (IC_50_: 1.18, 2.78, and 1.34 mg/mL for ABTS^•+^, hydroxyl radical, and superoxide anion radical scavenging assays, respectively) increased in proportion to the lower molecular weight.^44^ It has been proven that polysaccharide extracts tend to exhibit higher antioxidant activity compared to pure polysaccharides. The formation of polysaccharide complexes or conjugates with other components such as protein, amino acid, lipid, and peptide in polysaccharide extracts is particularly common, and these structures increase antioxidant properties.^31^ It has been suggested that the antioxidant activities of five different polysaccharide fractions obtained from *Cordyceps sinensis* were increased in direct proportion to their protein contents.^45^ Siu et al. reported that antioxidant activities of polysaccharide extracts obtained from *Trametes versicolor*, *Lentinula edodes*, and *Grifola frondosa* were correlated with increased protein contents with the amounts of 8.23 ± 0.12–25.1 ± 1.08, 0.63 ± 0.08–78.4 ± 1.07, and 0.55 ± 0.22–66.3 ± 0.45%, respectively.^46^*G. frondosa* polysaccharide extract with the highest protein content (78.4 ± 1.07%) was found to be the most antioxidant active in the study. *C. truncatus* and *M. procera* polysaccharide extracts were determined to be more active in terms of antioxidant activity. The higher antioxidant activity of both mushroom polysaccharide extracts may be due to their lower average MWs (2.27 × 10^3^ and 1.4 × 10^4^ Da for *C. truncatus* and 3.21 × 10^3^ and 1.21 × 10^4^ Da for *M. procera*) and higher total protein contents (2.85 ± 0.87% for *C. truncatus* and 3.78 ± 0.96% for *M. procera*).

**Table 4:** Antioxidant Activities of the Polysaccharide Extracts^a^

			polysaccharide extracts				standards			
			*C. tubaeformis*	*C. truncatus*	*H. pudorinus*	*M. procera*	α-tocopherol	BHA	ascorbic acid	EDTA
antioxidant activity	β-carotene-linoleic acid	inhibition (%)^b^	19.48 ± 1.14	56.91 ± 1.90	46.16 ± 1.28	41.43 ± 0.41	90.56 ± 0.79	92.93 ± 0.47	NT^e^	NT^e^
		IC_50_^c^	>800	472.16 ± 0.81	>800	>800	2.10 ± 0.08	1.34 ± 0.04	NT^e^	NT^e^
	DPPH^•^	inhibition (%)^b^	18.05 ± 0.18	11.71 ± 0.87	4.80 ± 0.17	39.03 ± 0.49	96.12 ± 0.42	97.79 ± 0.08	NT^e^	NT^e^
		IC_50_^c^	>800	>800	>800	>800	37.18 ± 0.41	19.76 ± 0.36	NT^e^	NT^e^
	ABTS^•+^	inhibition (%)^b^	20.93 ± 1.14	54.21 ± 0.96	15.65 ± 0.16	43.76 ± 0.76	94.96 ± 0.53	95.89 ± 0.10	NT^e^	NT^e^
		IC_50_^c^	>800	734.09 ± 0.43	>800	>800	38.46 ± 0.54	11.76 ± 0.09	NT^e^	NT^e^
	CUPRAC	absorbance^b^	0.59 ± 0.04	0.30 ± 0.01	0.37 ± 0.03	0.97 ± 0.04	2.93 ± 0.05	3.50 ± 0.04	NT^e^	NT^e^
		A_0.50_^c^	708.66 ± 0.79	>800	>800	387.50 ± 0.25	89.47 ± 0.87	24.42 ± 0.69	NT^e^	NT^e^
	PRAP	absorbance^b^	0.58 ± 0.01	0.25 ± 0.01	0.54 ± 0.01	0.97 ± 0.01	NT^e^	NT^e^	3.91 ± 0.01	NT^e^
		A_0.50_^c^	632.00 ± 0.01	>800	731.14 ± 0.01	384.08 ± 0.02	NT^e^	NT^e^	13.66 ± 0.01	NT^e^
	iron chelating assay	inhibition (%)^b^	69.99 ± 1.13	69.46 ± 0.11	76.02 ± 0.55	NA^d^	NT^e^	NT^e^	NT^e^	96.30 ± 0.11
		IC_50_^c^	496.98 ± 0.34	180.35 ± 0.59	208.84 ± 0.96	NA^d^	NT^e^	NT^e^	NT^e^	3.47 ± 0.14

a Values represent the means ± SE of three parallel sample measurements (*p* < 0.05).

b Results are given at 800 μg/mL concentration.

c Results are given as μg/mL.

d No activity.

e Not tested.

The antioxidant activities of *C. tubaeformis*, *C. truncatus*, and *H. pudorinus* polysaccharide extracts other than *M. procera* polysaccharide extract were first examined. *M. procera* (from China) polysaccharide extract was found to be weak antioxidant active in trolox equivalent antioxidant capacity (267.70 ± 16.06 μmol/g), FRAP (1.85 ± 0.28 mmol Fe/g), and metal chelating (143.50 ± 17.22 μmol Fe^2+^/g) methods.^47^ A significant antioxidant activity was observed in *M. procera* (from Bulgaria) polysaccharide in the oxygen radical absorbance capacity assay (313.3 ± 23.9 μmol TE/g) and no activity in the hydroxyl radical averting capacity assay.^23^*Macrolepiota dolichaula* polysaccharide was obtained as highly antioxidant active in hydroxyl radical scavenging (∼50% inhibition at 800 μg/mL), superoxide radical scavenging (∼90% inhibition at 300 μg/mL), and β-carotene-linoleic acid (∼50% inhibition at 400 μg/mL) assays in the study of Samanta et al.^48^ Antioxidant activity of *H. lucorum* was reported as weak by reducing power (absorbance: ∼0.01), hydroxyl radical scavenging (∼5% inhibition), DPPH^•^ scavenging (∼20% inhibition), and superoxide anion radical scavenging (∼15% inhibition) assays at 10.0 mg/mL.^39^ Antioxidant activities of CCPs-1 and CCPs-2 polysaccharides of *C. cornucopioides* were defined as moderate by using DPPH^•^ scavenging (EC_50_: 233.2 ± 14.4, 191.8 ± 19.5 μg/mL), reducing power (EC_50_: 210.5 ± 13.4, 190.1 ± 11.2 μg/mL), and metal chelating (EC_50_: 535.7 ± 45.7, 480.6 ± 17.8 μg/mL) assays.^18^ High DPPH^•^ (81.2% inhibition at 400 μg/mL) and ABTS^•+^ (99.4% inhibition at 500 μg/mL) scavenging activities were found in the *C. cornucopioides* polysaccharide fraction.^26^ The antioxidant activity results of the studied polysaccharide extracts and literature data supported each other.

### 2.7 Anticancer Activity

**Figure 4:**
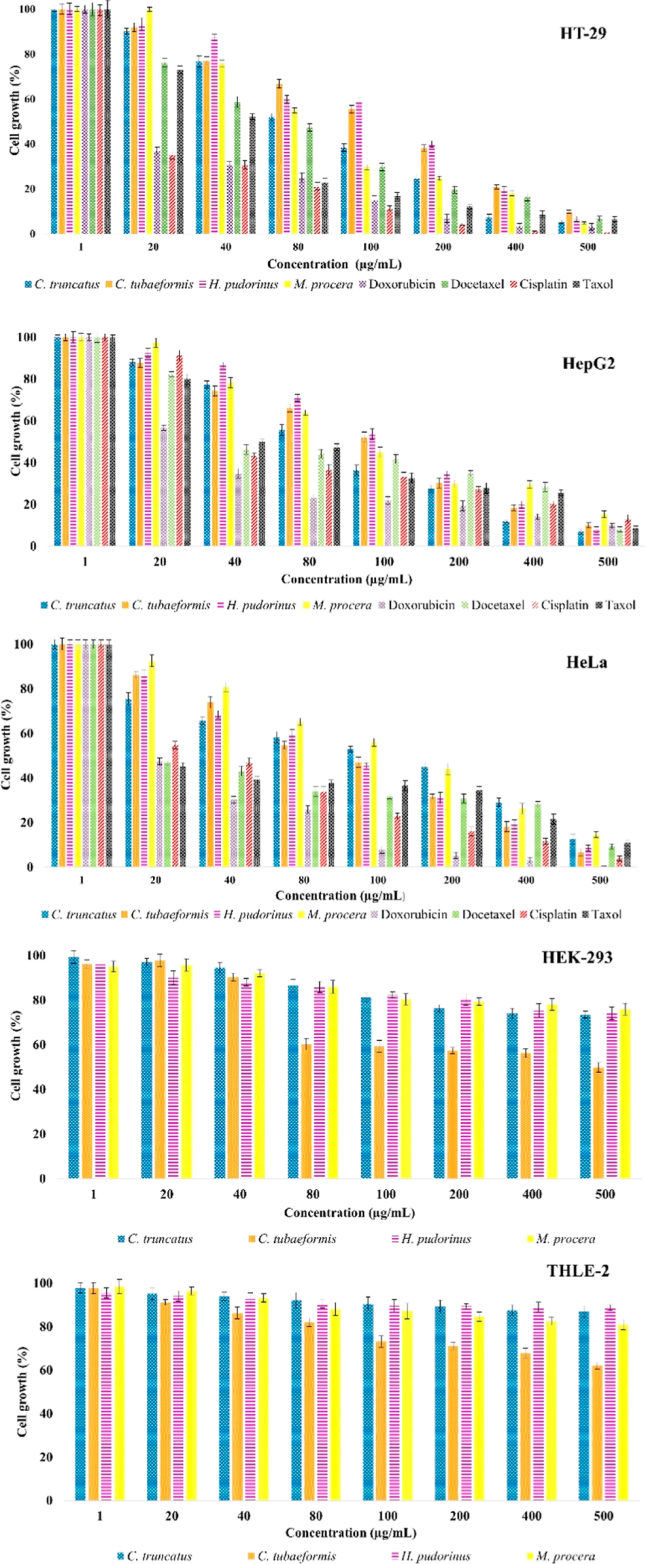
Cell growth values (%) of the polysaccharide extracts on HT-29, HepG2, HeLa, HEK-293, and THLE-2 cell lines. The error bars represent the means ± SE of three parallel measurements (*p* < 0.05).

**Figure 5:**
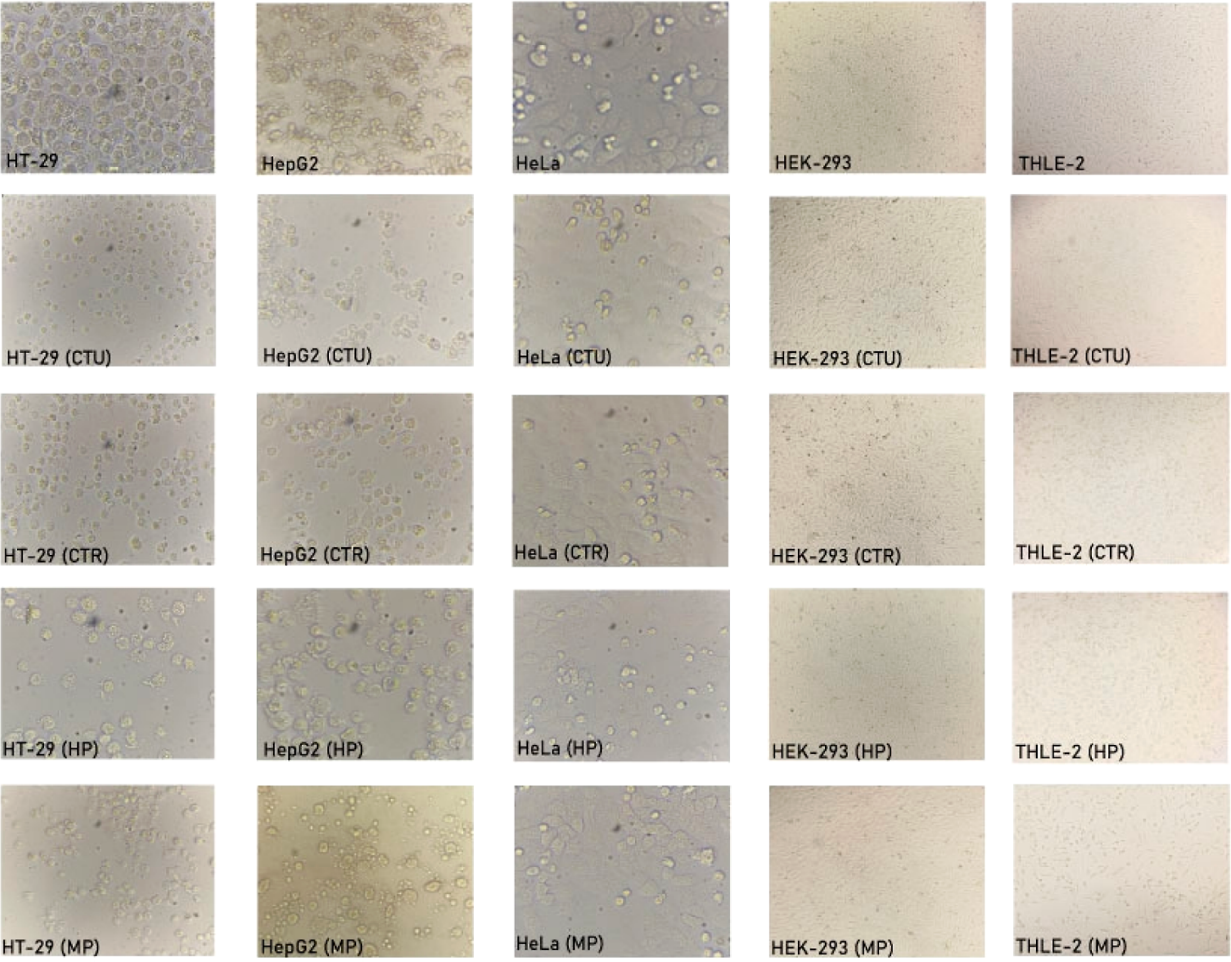
Morphological observations by the inverted microscope (10×) of cell lines treated with the polysaccharide extracts at 500 μg/mL concentration. CTU: *C. tubaeformis* polysaccharide extract, CTR: *C. truncatus* polysaccharide extract, HP: *H. pudorinus* polysaccharide extract, MP: *M. procera* polysaccharide extract.

The anticancer activities of the polysaccharide extracts were assayed on HT-29 (colon), HepG2 (liver), and HeLa (cervical) cell lines and HEK-293 (human embryonic kidney) and THLE-2 (liver epithelial) cell lines using the Alamar blue assay. The cell growth % values are given in Figure 4, and the IC_50_ results are in Table 5. The morphological observations of cell lines treated with the polysaccharide extracts are given in Figure 5. The best cell growth value % was recorded in *M. procera* polysaccharide extract as 4.94 ± 0.18% on HT-29 cell line, *C. truncatus* polysaccharide extract as 7.00 ± 0.40% on HepG2 cell line, and *H. pudorinus* polysaccharide extract as 6.64 ± 0.66% on HeLa cell line at 500 μg/mL. The cell growth values % were determined to be higher than 50% for all polysaccharide extracts on HEK-293 and THLE-2 cell lines. *C. truncatus* polysaccharide extract had the highest anticancer activity on HT-29 (IC_50_: 46.49 ± 0.26 μg/mL) and HepG2 (IC_50_: 48.50 ± 1.24 μg/mL) cell lines, while *H. pudorinus* polysaccharide extract had the highest anticancer activity on HeLa (IC_50_: 51.64 ± 0.26 μg/mL) cell line. It was noted that all polysaccharide extracts showed substantial anticancer activity against all three cancer cell lines. No cytotoxic activity was found on HEK-293 and THLE-2 cell lines in all polysaccharide extracts. The IC_50_ values of the standards doxorubicin, docetaxel, cisplatin, and taxol were found as follows: 15.56 ± 0.96, 29.90 ± 0.43, 14.75 ± 0.87, and 19.77 ± 1.04 μg/mL on HT-29 cell line; 11.36 ± 0.12, 31.33 ± 0.85, 27.35 ± 0.37, and 29.12 ± 0.14 μg/mL on HepG2 cell line; 19.78 ± 0.02, 28.80 ± 0.12, 31.02 ± 0.05, and 28.60 ± 1.04 μg/mL on HeLa cell line. The anticancer activities of the studied polysaccharide extracts were found to be lower compared to the standards.

**Table 5:** Anticancer Activities of Polysaccharide Extracts^a^,^b^

		polysaccharide extracts				standards			
		*C. tubaeformis*	*C. truncatus*	*H. pudorinus*	*M. procera*	doxorubicin	docetaxel	cisplatin	taxol
anticancer activity	HT-29	70.56 ± 1.41	46.49 ± 0.26	73.27 ± 0.59	47.67 ± 0.64	15.56 ± 0.96	29.90 ± 0.43	14.75 ± 0.87	19.77 ± 1.04
	HepG2	61.50 ± 0.96	48.50 ± 1.24	73.75 ± 0.77	64.37 ± 0.41	11.36 ± 0.12	31.33 ± 0.85	27.35 ± 0.37	29.12 ± 0.14
	HeLa	52.39 ± 0.45	56.29 ± 1.13	51.64 ± 0.26	77.87 ± 0.97	19.78 ± 0.02	28.80 ± 0.12	31.02 ± 0.05	28.60 ± 1.04
	HEK-293	>500	>500	>500	>500	NT^c^	NT^c^	NT^c^	NT^c^
	THLE-2	>500	>500	>500	>500	NT^c^	NT^c^	NT^c^	NT^c^

a Values represent the means ± SE of three parallel sample measurements (*p* < 0.05).

b Results are given as IC_50_ (μg/mL).

c Not tested.

It has been well described that the anticancer properties of polysaccharides are affected by various factors such as sugar units, main chain structures, glycosidic bond types, glycosidic bond configurations, degree of branching, three-dimensional structures, and MWs.^49^ Currently, the mechanisms of action of polysaccharides on cancer and the structures of the factors affecting this mechanism have not been fully elucidated.^50^ MW is an important factor in the anticancer activity of polysaccharides. It was stated that polysaccharides with a medium MW (20–500 kDa) showed significant anticancer activity, while polysaccharides with a smaller MW also possessed significant anticancer activity.^51^ Yang et al. showed that polysaccharide extracts with approximate MWs of 28 and 268 kDa obtained from *Flammulina velutipes* exhibited significant anticancer activity on BGC-823 (stomach) and A549 (lung) cell lines.^52^ It has been reported that two different polysaccharide extracts with approximate MWs of 5.6 × 10^4^ and 3.83 × 10^5^ Da obtained from *Sarcodon aspratus* displayed potent anticancer activity against HeLa cell line.^53^ Six different polysaccharide fractions with MWs <5 kDa from *Cerrena unicolor* were reported to indicate anticancer activity on HT-29 cell line.^54^ It has been suggested that the polysaccharide extract with an approximate MW of 9 kDa from *S. aspratus* indicated anticancer activity on HeLa cell line and induced apoptosis of cancer cells, in part through activation of caspase-3 and the mitochondrial pathway.^55^ These valuable anticancer properties of all studied polysaccharide extracts may be related to average MWs. In addition, the configuration of glycosidic bonds is also one of the important factors affecting the anticancer activity of polysaccharides. In general, the activity of α-glycosidic linked polysaccharides was stated as weaker, while the activity of β-glycosidic linked polysaccharides was stronger.^49,56^ Li et al. studied the anticancer activities of RVP-1 and RVP-2 polysaccharide extracts from *Russula virescens* against MCF-7 (breast), HepG2, and A549 (lung) cell lines and especially emphasized that RVP-1 with a greater proportion of β-glycosidic linked residue had better anticancer activity.^57^ Lentinan is a polysaccharide used clinically to improve immunity and prevent the proliferation of cancer cells with its (1 → 3)-β-d-glucopyranosyl main chain and (1 → 6)-β-glucosyl side chains.^50^*C. truncatus* polysaccharide extract showed the highest anticancer activity against HT-29 and HepG2 cell lines, and this may be related to the highest amounts of more β-configured residues (six residues) compared to other polysaccharide extracts.

The anticancer properties of the polysaccharide extracts in this study were revealed for the first time. In a previous study, the IC_50_ value of β-glucan type polysaccharide obtained from *Fomitopsis officinalis* in HeLa cell line was found to be 318 ± 47 μg/mL.^58^*Calocybe indica* polysaccharide extract was tested for anticancer activities on HeLa, HT-29, and HepG2 cell lines, and IC_50_ values were found to be 148.40, 151.00, and 168.30 μg/mL, respectively.^59^ Inhibition % values of HeLa cell line of *Pleurotus eryngii*, *Pleurotus nebrodensis*, *Pleurotus ostreatus*, *Hypsizygus marmoreus*, *F. velutipes*, *Lentinus edodes*, *G. lucidum*, and *Hericium erinaceus* were reported to vary in the range of 5–40% at 200 μg/mL.^60^ Moderate anticancer activity was defined in *Cordyceps militaris* polysaccharide (CMP-1) on HT-29 (IC_50_: 137.66 μg/mL), HeLa (IC_50_: 162.59 μg/mL), and HepG2 (IC_50_: 176.29 μg/mL) cell lines.^61^ It has been reported that *Sparassis crispa* polysaccharide extract had different degrees of effectiveness against Caco-2 (no activity), LS180 (IC_50_: 78 μg/mL), and HT-29 (IC_50_: 14 μg/mL) colon cancer cell lines.^62^ The cell viability values of *Ganoderma applanatum* polysaccharide extract on HeLa cell line were recorded as 62.46% at 50 μg/mL, 56.60% at 100 μg/mL, 51.90% at 200 μg/mL, 45.51% at 400 μg/mL, and 39.80% at 800 μg/mL.^63^ Compared with the aforementioned mushroom polysaccharides, the studied polysaccharide extracts showed strong anticancer activity on HT-29, HeLa, and HepG2 cell lines.

### 2.8 Enzyme Inhibition Activity

The enzyme inhibition activity of the polysaccharide extracts was examined against AChE, BChE, α-amylase, α-glucosidase, tyrosinase, and urease enzymes. The results are presented in Table 6. *H. pudorinus* polysaccharide extract was observed to be prominently active on AChE with an inhibition value of 49.14 ± 1.08% at 200 μg/mL and followed by *C. truncatus* polysaccharide extract (41.62 ± 1.18%). Only *C. truncatus* polysaccharide extract (4.70 ± 0.26%) demonstrated inhibition activity on BChE, while only *C. tubaeformis* polysaccharide extract (29.44 ± 0.94%) showed inhibition activity on urease at 200 μg/mL.

**Table 6:** Enzyme Inhibition Activities of the Polysaccharide Extracts^a^

polysaccharide extract						standards		
	*C. tubaeformis*	*C. truncatus*	*H. pudorinus*	*M. procera*	galantamine	thiourea	kojic acid	acarbose
AChE^b^	NA^d^	41.62 ± 1.18	49.14 ± 1.08	6.95 ± 0.19	78.76 ± 0.52	NT^e^	NT^e^	NT^e^
BChE^b^	NA^d^	4.70 ± 0.26	NA^d^	NA^d^	79.27 ± 0.56	NT^e^	NT^e^	NT^e^
urease^b^	29.44 ± 0.94	NA^d^	NA^d^	NA^d^	NT^e^	78.57 ± 0.22	NT^e^	NT^e^
tyrosinase^b^	8.09 ± 0.74	3.41 ± 0.20	8.71 ± 0.57	7.30 ± 0.69	NT^e^	NT^e^	47.81 ± 0.50	NT^e^
α-amylase^c^	1.87 ± 0.54	4.51 ± 0.20	6.00 ± 0.52	7.02 ± 0.19	NT^e^	NT^e^	NT^e^	89.57 ± 0.09
α-glucosidase^c^	31.32 ± 1.25	16.34 ± 0.75	28.60 ± 0.94	23.85 ± 0.95	NT^e^	NT^e^	NT^e^	67.01 ± 2.28

a Values represent the means ± SE of three parallel sample measurements (*p* < 0.05).

b Results are given as inhibition (%) at 200 μg/mL concentration.

c Results are given as inhibition (%) at 500 μg/mL concentration.

d NA: no activity.

e NT: not tested.

All polysaccharide extracts were found to be low active in tyrosinase (3.41 ± 0.20–8.71 ± 0.57% at 200 μg/mL) and α-amylase (1.87 ± 0.54–7.02 ± 0.19% at 500 μg/mL) inhibition assays. *C. tubaeformis* (31.32 ± 1.25%) and *H. pudorinus* (28.60 ± 0.94%) polysaccharide extracts were specified as the most active against α-glucosidase at 500 μg/mL. The standard galantamine indicated 78.76 ± 0.52 and 79.27 ± 0.56% inhibition on AChE and BChE at 200 μg/mL, respectively. The standard acarbose inhibited 89.57 ± 0.09% of α-amylase and 67.01 ± 2.28% of α-glucosidase at 500 μg/mL. The inhibition value of the standard thiourea on urease was 78.57 ± 0.22%, while the inhibition value of the standard kojic acid was 47.81 ± 0.50% on tyrosinase at 200 μg/mL. Generally, low enzyme inhibition activities were observed for all polysaccharide extracts compared to the standards. There is no proven mechanism of action in the literature regarding the ability to precisely regulate and determine the effects of polysaccharides on the inhibition of enzymes. Particularly, it has been presented that the properties of polysaccharide structures, such as mixed chemical structures, compositions, and configurations, cause limitations in interacting with the active sites of enzymes. In this regard, other bioactive components in the contents of polysaccharide extracts may interact with enzymes and cause enzyme inhibition.^64^ These varying enzyme inhibition activities of the studied polysaccharide extracts could be related to both their structural properties and other bioactive components.

In parallel with the results here, in the study of Xu et al., 14 mushroom polysaccharides were screened for tyrosinase inhibition activities.^47^ Among these investigated polysaccharide extracts, *M. procera* polysaccharide extract inhibited 10.15 ± 0.5% of tyrosinase, and *T. versicolor*, *Daedaleopsis sinensis*, *Daedaleopsis confragosa*, *Lenzites betulina*, *Armillaria ostoyae*, *Armillariella cepistipes*, *Lepista nuda*, *Russula foetens*, *Russula cyanoxantha*, *Russula persicina*, *Macrolepiota mastoidea*, *Handkea utriformis*, and *Chroogomphus rutilus* polysaccharide extracts inhibited 3.58 ± 0.18–84.68 ± 4.23% of tyrosinase at 1 mg/mL. We have described AChE (no activity, 10.56 ± 0.50%, 5.16 ± 1.04%, no activity, 21.14 ± 0.88%, 11.87 ± 0.62%, 8.62 ± 0.25%, and 29.32 ± 0.94%, respectively) and BChE (no activity, no activity, 27.63 ± 0.51%, 17.18 ± 0.17%, no activity, 56.31 ± 0.74% and no activity, respectively) inhibition activities of *Fomes fomentarius*, *Ganoderma adspersum*, *Fuscoporia torulosa*, *G. lucidum*, *Porodaedalea pini*, *Phellinus igniarius*, *G. applanatum*, and *P. ostreatus* polysaccharide extracts at 200 μg/mL.^28^ The IC_50_ values of *Coprinellus truncorum* and *Coprinus comatus* polysaccharide extracts were stated as 0.61 ± 0.03 and 0.62 ± 0.07 mg/mL in AChE inhibition activity assay.^65^ AChE (39.67 ± 2.11 and 20.54 ± 0.50%), BChE (48.22 ± 2.28 and 54.08 ± 2.88%), and tyrosinase (31.99 ± 2.32 and 31.99 ± 2.32%) inhibition activities of proteinized and deproteinized *Morchella esculenta* polysaccharides were reported at 100 μg/mL.^66^ Tyrosinase inhibition activities of hot-water extracted, microwave-assisted extracted, and ultrasonic-assisted extracted polysaccharide extracts of *Volvariella volvacea* were found to be 51.46, 34.88, and 34.17 mg KAE/g, respectively.^67^ In the study of Li et al., RVP-1 and RVP-2 polysaccharides from *R. virescens* showed high α-amylase (∼50, ∼75% inhibition) and α-glucosidase (77.59 and 77.41% inhibition) inhibitory activities at 3.2 mg/mL.^57^ α-Glucosidase inhibition values of HMP-1 and HMP polysaccharide extracts isolated from *H. marmoreus* were noted as 87.63 and 53.87% at 6 mg/mL, respectively.^68^ Galactomannan I and II polysaccharides from *G. adspersum* and *Rhizopogon luteolus* were investigated for AChE (IC_50_: > 50, 36.71 ± 0.94 μg/mL) and BChE (IC_50_: > 50, 40.18 ± 0.26 μg/mL) inhibition activities.^32^

## 3 Conclusions

In this study, *C. tubaeformis*, *C. truncatus*, *H. pudorinus,* and *M. procera* mushrooms were extracted by using ethanol precipitation from the hot water extraction method to obtain polysaccharide extracts. All polysaccharide extracts were chemically characterized and tested for antioxidant, anticancer, and enzyme inhibition activities. FT-IR and ^1^H NMR analyses confirmed the presence of characteristic carbohydrate patterns and proton interactions, suggesting that all polysaccharide extracts contain residues of α- and β-d-mannopyranose, d-glucopyranose, d-galactopyranose, α-l-arabinofuranose, and α-l-fucopyranose. Glucose and galactose were found to be the most abundant monosaccharides in all polysaccharide extracts by GC–MS. Additionally, the total carbohydrate and total protein contents and approximate MWs of the polysaccharide extracts were estimated. The polysaccharide extracts demonstrated moderate antioxidant activity and varying degrees of enzyme inhibition activity due to differences in their approximate MWs and chemical structures. In conjunction with the fact that all polysaccharide extracts had significant anticancer activity and did not show cytotoxic activity on liver epithelial and human embryonic kidney cell lines, these polysaccharide extracts are promising for evaluation in further *in vivo* and clinical studies, especially in comparison to the negative effects of cancer drugs on healthy cells.

In conclusion, this is the first study on *C. truncatus* and *H. pudorinus* polysaccharides. In addition, the antioxidant, enzyme inhibition, and anticancer activities of all obtained polysaccharide extracts were screened for the first time. The studied polysaccharide extracts, with their remarkable activities, enable further research on molecular structures and structure–activity relationships. The findings also support the applications of these polysaccharide extracts in nutraceutical foods. The fact that the obtained polysaccharides showed significant antioxidant and anticancer activities creates potential effects that can be an important starting point for new drug discovery in the pharmaceutical industry as a new alternative natural source in the treatment of many diseases caused by cancer and oxidative stress. Furthermore, the obtained results constitute an important bridge toward *in vitro* studies of mushroom polysaccharide extracts, especially for future clinical research on the development of antioxidant and anticancer drugs.

## 4 Materials and Methods

### 4.1 Mushroom Materials

*H. pudorinus* (Fr.) Fr., *C. tubaeformis* (Fr.) Quél., and *C. truncatus* Donk from Bolu-Turkey and *M. procera* (Scop.) Singer from Amasya-Turkey were collected in 2021. All mushroom species were identified by using Breitenbach and Kränzlin and Moser by Dr. Sinan Aktaş (Selçuk University, Konya, Turkey).^69,70^ The voucher specimens were deposited at the Fungarium of the Mushroom Research and Application Center of Selçuk University, Konya, Turkey. Voucher numbers: 5409 for *C. tubaeformis*, 5408 for *C. truncatus*, 3135 for *M. procera*, 5410 for *H. pudorinus*.

### 4.2 Chemicals

All used chemicals (analytical grade) were purchased from E. Merck (Darmstadt, Germany) and Sigma Chemical Co. (Sigma-Aldrich GmbH, Steinheim, Germany). These chemicals are as follows: fetal bovine serum (FBS), α-tocopherol, ethanol, EDTA, ascorbic acid, 2,2′-azino *bis* (3-ethylbenzothiazoline-6-sulfonic acid) diammonium salt (ABTS), methanol, hydrogen chloride, hexane, copper (II) chloride, Lugol solution, ferrous chloride, sodium carbonate, glucose, doxorubicin, 5,5′-dithio*bis*(2-nitrobenzoic) acid (DTNB), neocuproine, horse serum butyrylcholinesterase (BChE) (EC 3.1.1.8, 11.4 U/mg, Sigma, St. Louis, MO), BHA, penicillin streptomycin solution, starch, 1,1-diphenyl-2-picryl-hydrazyl (DPPH), cisplatin, 4-*N*-nitrophenyl-α-d-glucopyranoside (PNPG), rhamnose, trypsin–EDTA solution, standard dextrans, sodium chloride, xylose, 3-(2-pyridyl)-5,6-di (2-furyl)-1,2,4-triazine-5′,5″-disulfonic acid disodium salt (ferene), sodium phosphate, electric eel acetylcholinesterase (AChE) (type-VI-S, EC 3.1.1.7, 425.84 U/mg, Sigma, St. Louis, MO), mannose, Dulbecco’s modified eagle medium (DMEM), Coomassie brilliant blue G-250, arabinose, Jack Beans urease [type-III, EC 232-656-0, 20990 U/g solid], acetylthiocholine iodide, fucose, butyrylthiocholine chloride, galactose, Alamar Blue reagent, α-glucosidase from *Saccharomyces cerevisiae* (EC. 3.2.1.20), maltose, porcine pancreas α-amylase (EC. 3.2.1.1), docetaxel, sulfuric acid, sodium nitroprusside, cisplatin, acarbose, taxol, galantamine, ammonium molybdate, bovine serum albumin (BSA), and sodium hypochlorite.

### 4.3 Polysaccharides Extraction

The mushroom samples were air-dried in the darkness at room temperature, and the dried mushroom samples were first pulverized. The powdered fruiting bodies of mushroom samples were extracted with 80% ethanol at room temperature. After the ethanol extract was separated, the mushroom residue was dried and then extracted with hot water at 80 °C. After the water extract was filtered, 99% ethanol (4 times more than the filtrate volume) was added to the filtrate, and the polysaccharides were precipitated. Then, the precipitate was centrifuged at 4000 rpm for 15 min, and polysaccharide extracts were obtained from the mushrooms. The obtained polysaccharide extracts were dried using a freeze-dryer.^28^ All extracts were kept at +4 °C for a maximum of 10 days for further tests.

### 4.4 Total Carbohydrate and Total Protein Contents

The phenol-sulfuric acid method was used to test the total carbohydrate contents of the polysaccharide extracts.^71^ Glucose was used as the standard. The following equation, derived from the calibration curve, was used to calculate the total carbohydrate contents:
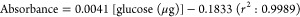


The Bradford method was used to test the total protein contents of the polysaccharide extracts.^72^ BSA was used as the standard. The following equation, derived from the calibration curve, was used to calculate total protein contents:
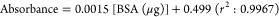


### 4.5 Determination of Monosaccharide Composition

The monosaccharide compositions of the polysaccharide extracts were analyzed by GC–MS (Varian Saturn 2100T, USA), as described in our earlier study.^28^ Seven sugar standards, such as galactose, fucose, rhamnose, mannose, xylose, arabinose, and glucose, were used for the identification of the monosaccharide compositions. 30 mg of polysaccharide extract was dissolved in water, 6 M TFA (trifluoroacetic acid) was added, and it was kept for 24 h at 100 °C. 300 μL of BSTFA (*N*,*O*-bis(trimethylsilyl) trifluoroacetamide) and 200 μL of pyridine were added to the hydrolysates and sugar standards and heated at 80 °C for 30 min to obtain trimethylsilyl derivatives. Sample derivatives were injected into GC-MS connected to HP-5 fused silica capillary column (30 m × 0.32 mm × 0.25 mm) after cooling. Chromatographic conditions were as follows: The carrier gas: He (flow rate: 1 mL/min); the injector temperature: 250 °C; the detector temperature: 270 °C; the initial column temperature was 100 °C for 5 min, increased progressively to 150 °C at 5 °C/min, and held at 150 °C for 5 min, then subsequently programmed as follows: 5 °C/min to 240 °C and held at 240 °C for 2 min. The relative molar ratios of monosaccharides were evaluated by the area normalization method according to GC–MS chromatograms.

### 4.6 FT-IR Analysis

FT-IR analyses of the polysaccharide extracts (5 mg) were performed with a Thermo Scientific Nicolet iS20 FT-IR instrument using attenuated total reflection supplied with a diamond crystal plate. The recorded spectra were the means of 32 spectra taken in the 500–4000 cm^–1^ wavelength range with a resolution of 0.5 cm^–1^ and atmospheric correction switched on at room temperature.

### 4.7 ^1^H NMR Analysis

Polysaccharide extracts were lyophilized three times to exchange with deuterium in D_2_O (99.9%). Then polysaccharide extracts were separately dissolved in D_2_O (99.9%) in a nuclear magnetic resonance (NMR) tube at a concentration of 60 mg/mL. ^1^H NMR analyses were achieved using the Agilent 600 MHz NMR instrument. The chemical shifts were stated in parts per million (ppm). The singlet resonance of trimethylsilane (TMS) at 0 ppm was internally referenced for spectra.

### 4.8 Determination of Approximate Molecular Weight

The approximate MWs of polysaccharides were determined by HPLC (Shimadzu LC-20 AT) connected to Shimadzu RID-10A detector (Shimadzu, Tokyo, Japan), as reported in our earlier study.^28^ The GPC Ultrahydrogel 1000 column (7.5 mm × 300 mm) was used for separation at 40 °C. 20 μL of the polysaccharide extract solution was injected, and the elution was carried out by using 0.05 M NaCl with a 0.5 mL/min flow rate. The approximate MWs of the polysaccharide extracts were calculated using a calibration curve of the logarithm of the molecular weight of the dextran standards versus elution volume. The approximate MWs of the polysaccharide extracts were calculated using the following equation derived from the calibration curve:
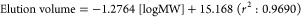


### 4.9 Antioxidant Activity

The antioxidant activity of the polysaccharide extracts was assayed by different *in vitro* assays, namely β-carotene-linoleic acid, ABTS^•+^ scavenging, PRAP, DPPH^•^ (1,1-diphenyl-2-picrylhydrazyl) scavenging, CUPRAC, and iron chelating assays.^73,74^ The effective concentration (IC_50_) displaying 50% percent inhibition was calculated using the graph of percent inhibition % versus concentration. The effective concentration (A_0.50_) displaying 0.500 absorbance was calculated using the graph of absorbance versus concentration. The results were given as percent inhibition % at 800 μg/mL and IC_50_ (μg/mL) values for β-carotene-linoleic acid, DPPH^•^ and ABTS^•+^ scavenging, and iron chelating assays, and absorbance at 800 μg/mL and A_0.50_ (μg/mL) values for CUPRAC and PRAP assays.

#### 4.9.1 β-Carotene-Linoleic Acid Activity

The total antioxidant activity of the polysaccharide extracts was tested by β-carotene-linoleic acid assay.^73^ β-carotene-linoleic acid mixture (160 μL) (linoleic acid, β-carotene, Tween 40) and the polysaccharide extract, control, or standard solution (40 μL) were mixed. Both the zero-time absorbance and after incubation for 2 h at 50 °C were measured at 470 nm.

#### 4.9.2 DPPH^•^ Scavenging Activity

The DPPH^•^ scavenging activity of the polysaccharide extracts was tested as expressed in our previous study.^73^ The polysaccharide extract, control, or standard solution (40 μL) and DPPH^•^ solution (160 μL) were mixed, and the absorbance was read at 517 nm after 30 min.

#### 4.9.3 ABTS^•+^ Scavenging Activity

The ABTS^•+^ scavenging activity of the polysaccharide extracts was tested as expressed in our previous study.^73^ The polysaccharide extract, control, or standard solution (40 μL) and ABTS^•+^ solution (160 μL) were mixed, and the absorbance was read at 734 nm after 10 min.

#### 4.9.4 Cupric Reducing Antioxidant Capacity Activity

The CUPRAC activity of the polysaccharide extracts was tested as expressed in our previous study.^73^ CuCl_2_ (50 μL), NH_4_Ac buffer (60 μL), neocuproine (50 μL), and the polysaccharide extract, control, or standard solution (40 μL) were mixed. The absorbance was read at 450 nm after 1 h.

#### 4.9.5 Metal Chelating Activity

The metal chelating activity of the polysaccharide extracts was tested, as expressed in our previous study.^73^ The polysaccharide extract, control, or standard solution (80 μL), FeCl_2_ (40 μL), and ferene (80 μL) were mixed, and the absorbance was read at 593 nm.

#### 4.9.6 Phosphomolybdenum Reducing Antioxidant Power

The PRAP activity of the polysaccharide extracts was tested as expressed in the study of Prieto et al.^74^ The polysaccharide extract, control, or standard solution (300 μL) and the reagent solution (3 mL) [H_2_SO_4_, Na_3_PO_4_, (NH_4_)_6_Mo_7_O_24_] were incubated for 90 min at 95 °C. When the mixture cooled to room temperature, the absorbance was read at 695 nm.

### 4.10 Anticancer Activity

Anticancer activity of the polysaccharide extracts was assayed on HT-29 (colon), HepG2 (liver), and HeLa (cervical) cell lines and HEK-293 (human embryonic kidney) and THLE-2 (liver epithelial) cell lines using the Alamar blue assay.^75^ Cells kept at −80 °C were thawed in a 37 °C water bath, centrifuged, and then transferred to the growth medium. Then, the cells were incubated in DMEM (10% FBS, 1% penicillin–streptomycin, and 0.01% gentamicin) and RPMI (10% FBS, 1% penicillin–streptomycin, and 0.01% gentamicin) mediums at 37 °C in a 5% CO_2_ atmosphere. When the active cells reached sufficient capacity, they were placed in transition media, washed with phosphate-buffered saline, and separated from the surface according to the trypsinization method. A dilution of the resulting cell pellets was made in an appropriate medium, and cell pellets were placed in cell culture dishes, including fresh medium. Anticancer activity was tested according to the Alamar Blue assay. Cell lines were seeded in 96-well plates and incubated at 37 °C and 5% CO_2_. After the growth medium was removed, polysaccharide extract, control, or standard were attached to each well, and after 18 h, Alamar Blue reagent was added and incubated for 4 h. Absorbance was read at 570 and 600 nm. The results were presented as cell growth % and effective concentration displaying 50% inhibition percent (IC_50_ μg/mL). The morphology of the cell lines-treated with the polysaccharide extracts were observed by inverted microscopy.

### 4.11 Enzyme Inhibition Activity

Th inhibition activity of the polysaccharide extracts was assayed on AChE (acetylcholinesterase), BChE (butyrylcholinesterase), α-amylase, α-glucosidase, tyrosinase, and urease enzymes.^76^ The results were given as percent inhibition % (at 200 μg/mL for AChE, BChE, tyrosinase, and urease and at 500 μg/mL for α-amylase, and α-glucosidase) and IC_50_ values.

#### 4.11.1 AChE and BChE Inhibition Activity

The AChE and BChE inhibition activities of the polysaccharide extracts were tested by the Ellman method.^76^ The polysaccharide extract, control, or standard solution (10 μL), sodium phosphate buffer (130 μL), AChE, or BChE in buffer (20 μL) were mixed and incubated at 25 °C for 15 min. Then, DTNB (20 μL) and acetylthiocholine iodide or butyrylthiocholine chloride (20 μL) were added. The absorbance was read at 412 nm.

#### 4.11.2 α-Amylase and α-Glucosidase Inhibition Activity

The α-amylase and α-glucosidase inhibition activities of the polysaccharide extracts were tested as expressed in our previous study.^76^ For the α-amylase inhibition assay, the polysaccharide extract, control, or standard solution (25 μL) and α-amylase in phosphate buffer (50 μL) were first incubated at 37 °C for 10 min. Then, starch solution (50 μL) was added and incubated at 37 °C for 10 min. Lugol solution (100 μL) and HCl (25 μL) were added, and the absorbance was read at 565 nm.

For the α-glucosidase inhibition assay, the polysaccharide extract, control, or standard solution (10 μL), phosphate buffer (50 μL), PNPG in phosphate buffer (25 μL), and α-glucosidase in phosphate buffer (25 μL) were incubated for 20 min at 37 °C. Then, Na_2_CO_3_ (90 μL) was added, and the absorbance was read at 400 nm.

#### 4.11.3 Urease Inhibition Activity

The urease inhibition activity of the polysaccharide extracts was tested by an indophenol test.^76^ The polysaccharide extract, control, or standard solution (10 μL), urease in buffer (25 μL), and urea in buffer (50 μL) were mixed and incubated for 15 min at 30 °C. Then, alkali reagent (70 μL) (0.5% NaOH and 0.1% NaOCl) and phenol reagent (45 μL) (1% phenol and 0.005% sodium nitroprusside) were added. The absorbance was read at 630 nm.

#### 4.11.4 Tyrosinase Inhibition Activity

The polysaccharide extracts were tested for tyrosinase inhibition activity.^76^ Sodium phosphate buffer (150 μL), the polysaccharide extract, control, or standard solution (50 μL), and tyrosinase in buffer (20 μL) were mixed and incubated at 37 °C for 10 min; then l-DOPA (50 μL) was added. The absorbance was read at 475 nm after 10 min incubation at 37 °C.

### 4.12 Statistical Analysis

All results were presented as the mean ± SE (standard error) of three replicates. The estimation of differences in comparison of means was based on Student’s *t*-test, and *p* < 0.05 values were recorded as significant.
